# Malignant ovarian mixed germ cell tumour: a rare combination

**DOI:** 10.2349/biij.1.2.e10

**Published:** 2005-10-01

**Authors:** M Koshy, A Vijayananthan, V Vadiveloo

**Affiliations:** 1 Department of Biomedical Imaging (Radiology), Faculty of Medicine, University of Malaya, Kuala Lumpur, Malaysia; 2 Department of Obstetrics and Gynaecology, Tengku Ampuan Rahimah Hospital, Klang, Selangor, Malaysia

**Keywords:** Malignant mixed germ cell tumour, embryonal carcinoma, choriocarcinoma

## Abstract

Ovarian germ cell tumours are very rare and affect mainly young girls and women. Due to this, the conservation of reproductive potential is of great concern. One of the most remarkable advances in oncology is in the treatment of malignant ovarian germ cell tumours. Two histological groups are distinguished: dygerminomas, equivalent to testicular seminomas, and non-dysgerminomatous tumours. We report a case of a 30-year-old nulliparous woman who presented with persistent per vaginal bleeding and was found to have a malignant mixed germ cell tumour comprising of both embryonal carcinoma and choriocarcinoma.

## INTRODUCTION

Ovarian germ cell tumours comprise approximately 15% to 20% of all ovarian neoplasms. They are rapidly growing neoplasms that arise from primordial germ cells derived from the embryonal gonad. Malignant germ cell tumours comprise less than 5% of all ovarian neoplasms. The incidence of malignant ovarian germ cell tumours range from 1 to 6 percent as reported in the West and from 8 to 19 percent in Asia [1]. Two histological groups are distinguished: dygerminomas, equivalent to testicular seminomas, and non-dysgerminomatous tumours.

The diagnosis suspected on physical examination, relies on pelvic or transvaginal ultrasonography detection of a voluminous ovarian mass responsible for abdominal discomfort and/or swelling. However, this is only confirmed during the initial surgical intervention. Tumour markers such as human chorionic gonadotropin (HCG) and alpha-fetoprotein also contribute to the diagnosis, the prognosis and follow-up of the disease.

We present a case of malignant mixed germ cell tumour comprising of embryonal carcinoma and choriocarcinoma. The term “embryonal carcinoma” was used in a study by Kurman *et al.*, as it is analogous to embryonal carcinoma of the adult testis and can be distinguished from the endodermal sinus tumour by its distinctive morphology, immunohistochemistry, and clinical features [2].

## CASE REPORT

A 30-year-old nulliparous woman had significant persistent per vaginal bleeding and was seen by a private obstetrician. An ultrasound of the pelvis was done and it was said to be normal at that time. She was treated with progestogens for four cycles and her menstrual cycle resumed to being normal. Six months later she presented again with per vaginal bleeding and a repeat ultrasound revealed a pelvic mass. A diagnostic laparoscopy was then done and deposits were found in both adnexa but no biopsy was taken. She was once again treated with progestogens for two cycles.

One month later she presented again with per vaginal spotting for four weeks and was then referred to the gynaecology clinic at University Malaya Medical Centre. She also complained of lower abdominal pain but denied any other symptoms.

Physical examination revealed that she had slight pallor and was afebrile. Vital signs were as follows: blood pressure of 107/72 mmHg, pulse rate 114 bpm, respiration 18/min. She had a distended abdomen with a firm, non-pulsatile, palpable mass in the pelvis and extending up to just below the umbilicus. There was mild periumbilical tenderness, but no guarding or rebound tenderness.

Rectal examination was negative for occult blood, and there were no masses felt. There was no inguinal adenopathy. Past history revealed that she had experienced menarche at age 12. Her menstrual periods were described as regular cycles with normal flow. There was no family history of any gynaecological cancer.

**Figure 1:**
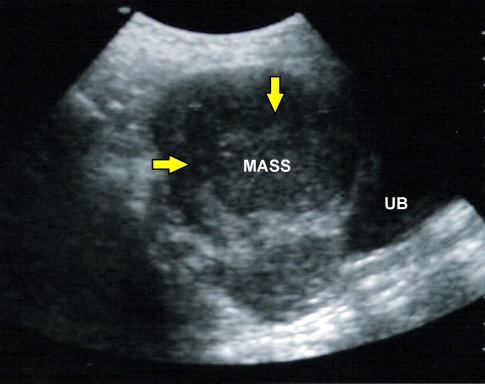
Ultrasound examination revealed a mass of mixed echogenicity in the right adnexa (arrows). UB = urinary bladder.

**Figure 2:**
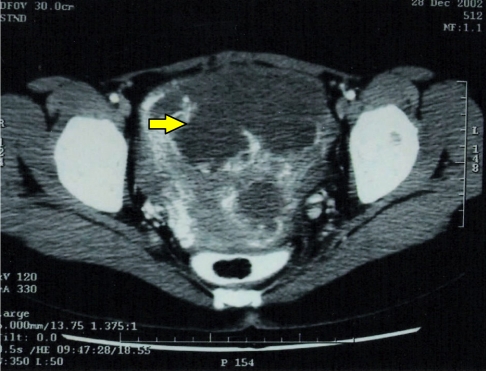
Computed tomography scan of the pelvis revealed a highly vascular enhancing mass (arrow) in the pelvis.

Initial laboratory results revealed a normal complete blood count, electrolytes, blood urea, creatinine and glucose, but a low haemoglobin count of 91.6 g/L. Her urine pregnancy test was positive. The patient underwent exploratory laparotomy for suspected ectopic pregnancy, as ultrasound scan showed an enlarged uterus with no gestational sac and a right heterogeneous ovarian mass measuring 4 cm x 5cm x 2.5cm (Figure 1). A right ovarian tumour with deposits on the uterus was found (Figure 2). Right cystectomy was then performed and a biopsy of the uterine deposits was taken.

**Figure 3:**
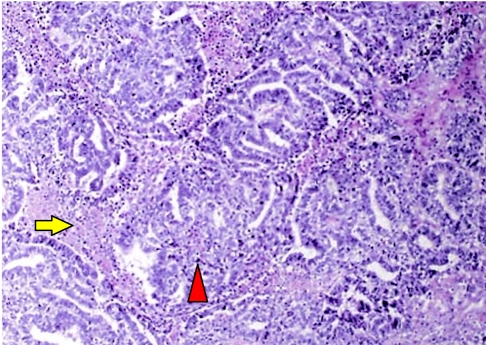
Photomicroscopy of mixed germ cell tumour showing a combination of embryonal carcinoma (arrowhead) and choriocarcinoma as evidenced by the presence of a sheet of cytotrophoblasts surrounded by syncytiotrophoblasts (arrow) elements.

On gross pathologic examination, sections through the ovary showed ‘a very haemorrhagic and necrotic tumour’. Microscopic specimens determined the tumour to be a germ cell tumour with embryonal carcinoma and choriocarcinoma elements (Figure 3).

**Figure 4:**
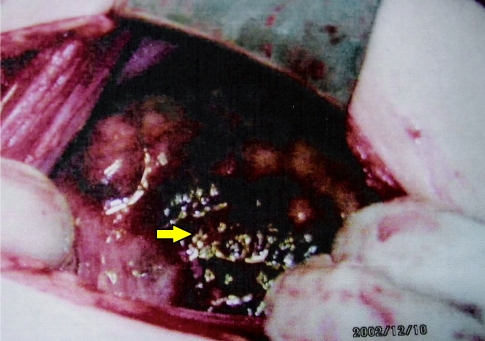
Intraoperative findings showing deposits (arrow) on the uterine surface.

A thoracic, abdominal and pelvic computed tomography (CT) scan was then obtained to stage the tumour. CT scan showed a highly vascular enhancing mass in the pelvis measuring 11.4cm × 11.3cm × 11.0cm (Figure 4) with multiple hypodense lesions in the liver and pancreas. There was no abdominal lymphadenopathy. Multiple hyperdense nodules of various sizes were seen throughout both lung fields and an enlarged pretracheal lymph node was also evident. The tumour was classified as Stage IV.

Despite multiple chemotherapy regimes, serial ultrasound five months later showed an enlarged uterus measuring 10.0cm × 6.6cm × 9.0cm with two hypodense areas within it and a 2.8cm × 4.7cm mass in the pouch of Douglas.

Six months later patient presented to the emergency department with headache and unsteady gait for two days. Computed tomography of the brain showed hemorrhagic cerebellar metastases. The patients conditioned gradually deteriorated and she succumbed to her illness two weeks later.

## DISCUSSION

Ovarian germ cell neoplasms are thought to be derived from primitive germ cells of the embryonic gonad. They constitute the second largest group accounting for 15 to 20 percent of all ovarian neoplasms. Malignant germ cell tumours comprise less than 5 percent of all ovarian neoplasms [3]. In 1973, the World Health Organization classified germ cell tumours as Dysgerminoma, Endodermal sinus tumour, Embryonal carcinoma, Polyembryoma, Choriocarcinoma, Teratomas (Immature, Mature, Monodermal), Mixed and Gonadoblastoma [4]. This initiative represented a major advance in terms of standardisation of nomenclature and histological criteria.

These tumours can occur in women at any age, but peak incidence is seen during the early 20’s. In children and adolescents, more than 60% of ovarian neoplasms are of germ cell origin, of which approximately 1/3 are malignant. In adults, the vast majority of germ cell tumours are benign (nearly all mature cystic teratomas).

Dysgerminoma is the most common germ cell tumour, accounting for 50% of all germ cell tumour cases. The yolk sac tumour (also known as endodermal sinus tumour) is the second most common germ cell tumour, accounting for 20% of all cases, and is common in girls and young adults with an average age of 19 years. Less common germ cell tumours are embryonal carcinoma, immature teratoma, choriocarcinoma, polyembryomas, and mixed germ cell tumours.

Embryonal carcinoma of the ovary is an extremely rare tumour and represents only about 4 per cent of malignant ovarian germ cell tumours [5]. It is distinguished from a choriocarcinoma by the absence of syncytiotrophoblastic and cytotrophoblastic cells [6]. Pure nongestational choriocarcinoma of the ovary is extremely rare, as it is nearly always admixed with other germ cell elements [2]. Histologically, it has the same appearance as gestational choriocarcinoma which metastasize to the ovaries [6]. Mixed germ-cell tumours of the ovary contain two or more elements of the lesions described above. In our case the combination was that of embryonal carcinoma and choriocarcinoma.

Clinically, a substantial majority of patients with germ cell tumours present with abdominal pain, abdominal distension or a pelvic mass. Approximately 10 percent of patients will present with acute abdominal pain, usually caused by rupture, haemorrhage, or torsion of the ovarian mass. A few patients will exhibit isosexual precocity, presumably due to HCG production by the tumour [7]. Embryonal carcinomas may secrete estrogens, with the patient exhibiting symptoms and signs of precocious pseudopuberty or irregular bleeding. In the case of non-gestational choriocarcinoma the presence of high HCG levels causes isosexual precocity which has been seen to occur in about 50 percent of patients whose lesions appear before menarche. In our case, the patient presented with abdominal distension and irregular bleeding but there was no evidence of precocious puberty.

Both HCG and alpha-fetoprotein are secreted by some germ cell malignancies, therefore the presence of circulating hormones may prove to be useful in the diagnosis and in monitoring the response to treatment [5,6]. Ultrasonography or CT is helpful in delineating the size and complexity of these tumours. Pre-operative staging of tumour is possible with CT [1].

Microscopically a dysgerminoma component is present in 80 percent, endodermal sinus tumour in 70 percent, immature teratoma in 53 percent, choriocarcinoma in 20 percent and embryonal carcinoma in 16 percent. A mixture of dysgerminoma and endodermal sinus tumour is the most common combination, accounting for one-third of the cases [2]. The immunohistochemical identification of HCG in syncytiotrophoblastic cells indicates a close relationship of embryonal carcinoma to choriocarcinoma [4].

Treatment consists of salpingo-oophorectomy with adjunctive chemotherapy. Chemotherapeutic regimens have evolved to combination therapy with overall disease- free survival rates of greater than 95 percent [4]. Due to relatively low toxicity and ease of treatment, chemotherapy has replaced radiation as the preferred surgical adjuvant even when fertility is not an issue [7]. Second-look laparotomy was earlier incorporated into the routine management of patients with epithelial ovarian cancer to assess disease status after a fixed interval of chemotherapy. However a study conducted by Gynaecologic Oncology Group (GOG) show rather conclusively that second-look laparatomy is not necessary in patients with tumour completely resected initially or in those patients with initially incompletely resected tumour that does not contain teratoma [4,7]. In view of malignant germ cell tumours occurring almost exclusively in young females, preservation of their ovarian function and fertility is becoming an important, although controversial, issue in gynaecologic oncology. A study by Zanetta *et al.* confirmed that normal gonadal function and fertility are possible after conservative surgery for ovarian germ cell malignancies, even with adjuvant chemotherapy [8].

Mixed germ cell tumour (embryonal carcinoma and choriocarcinoma) is a very rare tumour. Orientals may have a higher proportion of non-dysgerminomatous malignant ovarian germ cell tumours when compared to reports in the Western literature [1]. Careful initial surgery with adequate staging biopsies followed by combination chemotherapy can greatly improve the prognosis of these patients [3].
